# Safety and efficacy of stenting for symptomatic intracranial artery stenosis: a systematic reveiw and meta-analysis

**DOI:** 10.3389/fphar.2023.1122842

**Published:** 2023-06-08

**Authors:** Ting Shi, ShiJian Chen, YongPei Long, ZhongDeng Gu

**Affiliations:** ^1^ The Department of Blood Transfusion, Yongchuan Hospital, Chongqing Medical University, Chongqing, China; ^2^ The Department of Clinical Laboratory Medicine, Yongchuan Hospital, Chongqing Medical University, Chongqing, China; ^3^ The Department of Rehabilitation, Yongchuan Hospital, Chongqing Medical University, Chongqing, China

**Keywords:** symptomatic intracranial artery stenosis, stenting, systematic review, aggressive medical management, safety and efficacy

## Abstract

**Background:** Stroke is currently the second-leading cause of death just behind ischaemic heart disease. Drug therapy is currently the standard of care for patients with symptomatic intracranial artery stenosis (sICAS). Stenting is an important treatment for the prevention and treatment of ischemic stroke. It has been suggested that vertebral artery stenting might reduce this risk, but operation-related complications limit the application of stenting in the treatment of ischemic stroke. The differences in the safety and efficacy of stenting combined with drugs and drugs alone in the treatment of sICAS are unclear. The aim of this study was to assess the impact of both treatment modalities on the prognosis of patients with sICAS through a systematic review and meta-analysis.

**Methods:** The Chinese databases (CNKI, Wanfang, VIP, CBM, DUXIU) and English databases (Pubmed, Embase, Ovid_medline, Cochrane library, Web of science)were searched to identify all studies describing sICAS. The “Risk of Bias Assessment” tool and the “Jadad Scale” provided by the Cochrane Collaboration were used to evaluate the risk of bias and quality of the collected literature. The risk ratio (RR) and its 95% confidence interval (CI) were determined using Stata statistical software version 14.0.

**Results:** A total of 11 studies were included, comprising a total of 1,915 patients. The combined results of the study showed no significant difference between the incidence of transient cerebral ischemia (TIA)and stroke in patients with sICAS treated with drugs in combination with stents versus drugs alone. The incidence of death or stroke, cerebral haemorrhage, disabling stroke or death was significantly higher in patients receiving stent-combined drug therapy versus drug therapy alone for sICAS.

**Conclusion:** Studies suggest that stenting combined with medication for patients with sICAS may increase the incidence of death or stroke, cerebral haemorrhage, stroke or death, but has no significant effect on the incidence of TIA and stroke. The studies report inadequate and conflicting data and therefore the safety and efficacy of stenting for sICAS should be interpreted with caution.

**Systematic Review Registration:** https://www.crd.york.ac.uk/prospero/display_record.php?ID=CRD42022377090, identifier CRD42022377090

## 1 Introduction

Stroke is currently the second-leading cause of death just behind ischaemic heart disease. Atherosclerotic intracranial artery stenosis (sICAS), one of most common causes of stroke, accounted for 10%–54% (that reflects differences in prevalence in Western and Eastern societies rather than random variability.) of all ischaemic strokes. This is particularly true for patients with stroke or transient cerebral ischaemia (TIA) with moderate stenosis, where the 1-year stroke recurrence rate is as high as 23% (Zaidat et al., 2015). Drug therapy is currently the standard of care for patients with sICAS (Wang et al., 2018). Stenting is an important treatment for the prevention and treatment of ischemic stroke. It has been suggested that vertebral artery stenting might reduce this risk (Markus et al., 2019), but operation-related complications limit the application of stenting in the treatment of ischemic stroke (Si et al., 2022). Early studies concluded with acceptable perioperative complication rates and potential benefits (Wong et al., 2010; Turan et al., 2014). Aggressive medical management (i.e., dual antiplatelet therapy along with intensive modifiable risk factor management) is supported by the latest studies (Derdeyn et al., 2014; Leung et al., 2015; Zaidat et al., 2015). The differences in the safety and efficacy of stenting combined with drugs and drugs alone in the treatment of sICAS are unclear. The aim of this study was to assess the impact of both treatment modalities on the prognosis of patients with sICAS through a systematic review and meta-analysis.

## 2 Methods

### 2.1 Search strategy

The Chinese databases (CNKI, Wanfang, VIP, CBM, DUXIU) and English databases (Pubmed, Embase, Ovid_medline, Cochrane library, Web of science) were searched to identify all studies describing sICAS. The search terms used were related to the following key words: “Intracranial artery stenosis,” “Intracranial atherosclerosis,” “Cerebral Infarctions,” “stenting”. The search string is included in detail in Appendix A. The search was completed on 5 November 2022 and updated on 5 December 2022.

### 2.2 Inclusion, exclusion and quality evaluation

The inclusion criteria were as follows: 1. Randomised controlled trials (RCTs); 2. Patients with a clinical diagnosis of sICAS; 3. experimental groups treated with stenting combined with pharmacotherapy and control group with pharmacotherapy alone; 4. Outcomes include defined primary or secondary outcome indicators.

The exclusion criteria were as follows: 1. Patients with extracranial stenosis; 2. Outcome variables not reported; 3. Reviews, animal studies.

### 2.3 Assignments

Two authors (TS and SC) independently extracted data from eligible studies and used the Jadad scoring scale (Jadad et al., 1996) for quality assessment and the study was reported in accordance with the Preferred Reporting Items for Systematic Reviews and Meta-Analysis (PRISMA) guidelines (Page et al., 2021). When two investigators disagreed, a third investigator (ZG) was asked to decide on eligibility. Scoring items included mainly random sequence generation (0–2 points), blinding (0–2 points), allocation and concealment (0–2 points), and withdrawal or loss to follow-up (0-1 point). The evaluation score is shown in Table 1.

**TABLE 1:** Characteristics of the studies included in the meta-analysis.

First author year	Country	Participant (Stenting/medication) (N)	Maternal mean age (SD)	Male (%)	Type of stenting	Enroll period	Hypertensi on (%)	Dyslipide mia (%)	Diabetes mellitus (%)	Coronary artery disease (%)	Smoker (never/previous/current) (%)	Stenosis	Doses of medication	Quality assessment
Derdeyn et al., 2013 (Leung et al., 2015)	United States	451 (224/227)	a (61.0 ± 10.7)/b (59.5 ± 11.8)	a (56.7)/b (63.9)	Wingspan	2008–2011	a (89.7)/b (89.4)	a (86.6)/b (89.4)	a (47.3)/b (45.4)	a (21.0)/b (26.0)	a (40.4/65.4/24.2)b (34.4/35.2/30.4)	≥70	Aspirin 325 mg daily (Long-term use) Clopidogrel 75 m daily (90 days)	7
Zaidat et al., 2015 (Jadad et al., 1996)	United States	112 (59/53)	a (61.8 ± 12.3)/b (61.8 ± 12.8)	a (70.7)/b (60.4)	unlear	2009–2012	a (84.5)/b (81.1)	a (50.0)/b (60.4)	a (43.1)/b (37.7)	a (17.2)/b (22.6)	a (43.1/37.9/19.0)b (45.3/32.1/22.6)	≥70	Aspirin 81–325 mg daily (Long-term use) Clopidogrel 75 mg daily (90 days)	7
Peng Gao et al., 2022 (Page et al., 2021)	China	358 (176/182)	a (56.7 ± 9.4)/b (55.9 ± 9.8)	a (72.7)/b (74.2)	Wingspan	2014–2016	a (66.5)/b (68.7)	a (10.2)/b (11.5)	a (32.4)/b (24.2)	a (10.8)/b (10.4)	a (54.5/22.2/23.3)b (51.6/20.9/27.5)	≥70	Aspirin 100 mg daily (Long-term use) Clopidogrel 75 mg daily (90 days)	7
Zhongrong Miao et al., 2012 (Derdeyn et al., 2014)	China	70 (36/34)	a (53.42 ± 13.55)/b (49.18 ± 9.29)	a (66.7)/b (73.5)	Multiple sents	2007–2010	unlear	unlear	unlear	unlear	unlear	≥70	Aspirin 100 mg daily (Long-term use) Clopidogrel 75 mg daily (90 days)	6
Marc I. Chimowitz et al., 2011 (Zaidat et al., 2015)	United States	451 (224/227)	a (53.42 ± 13.55)b/(49.18 ± 9.29)	a (56.7)/b (63.9)	Wingspan	2008–2011	a (89.7)/b (89.4)	a (86.6)/b (89.4)	a (47.3)/b (45.4)	a (21.0)/b (26.0)	a (40.4/65.4/24.2)b (34.4/35.2/30.4)	≥70	Aspirin 325 mg daily (Long-term use) Clopidogrel 75 mg daily (90 days)	7
Ma et al., 2018 (Gao et al., 2022)	China	60 (30/30)	a (63.98 ± 4.45)/b (63.45 ± 4.38)	a (53.3)/b (60.0)	Wallstent	2016–2017	unlear	unlear	unlear	unlear	unlear	unlear	Aspirin 100 mg daily (Long-term use) Clopidogrel 75 mg daily (180 days)	5
Ding et al., 2021 (Miao et al., 2012)	China	70 (35/35)	a (56.7 ± 6.5)/b (57.2 ± 6.7)	a (60.0)/b (54.2)	unlear	2015–2019	a (57.1)/b (51.4)	a (14.3)/b (20.0)	a (25.0)/b (20.0)	unlear	unlear	unlear	Aspirin 100 mg daily (Long-term use) Clopidogrel 75 mg daily (90 days)	4
Huang et al., 2022 (Chimowitz et al., 2011)	China	142 (71/71)	a (61.09 ± 4.31)/b (60.28 ± 4.28)	a (52.1)/b (53.5)	Gateway	2018–2020	unlear	unlear	unlear	unlear	unlear	unlear	Aspirin 150 mg daily (10 days)	5
Zhu et al., 2011 (Ma, 2018)	China	71 (32/39)	a (56.56 ± 12.50)/b (50.46 ± 11.43)	a (65.6)/b (74.3)	unlear	2007–2010	a (37.5)/b (51.2)	a (75.0)/b (61.5)	a (84.3)/b (79.4)	a (93.7)/b (82.0)	unlear	≥70	Aspirin 100 mg daily (Long-term use) Clopidogrel 75 mg daily (90 days)	4
Li et al., 2013 (Ding et al., 2021)	China	96 (48/48)	a (56.3 ± 11.8)/b (57.1 ± 12.1)	a (52.0)/b (56.2)	unlear	2006–2010	unlear	unlear	unlear	unlear	unlear	unlear	Aspirin Ozagrey (Dose not mentioned)	4
Gao et al., 2013 (Huang et al., 2022)	China	34 (16/18)	unlear	unlear	unlear	2010–2011	unlear	unlear	unlear	unlear	unlear	unlear	Aspirin 100 mg daily (Long-term use) Clopidogrel 75 mg daily (90 days)	5

Study data extracted by (TS), including first author, year of publication, country, sample size, gender, age, stent type, follow-up time, hypertension, lipid metabolism disorders, diabetes, coronary heart disease, smoking history, and degree of stenosis.

### 2.4 Research indicators

The following indicators were observed in this study: 1. TIA and stroke rates; 2. Any stroke and mortality rates 3. The rate of cerebral haemorrhage 4. The rate of disabling strokes and mortality.

### 2.5 Statistical analysis

The risk ratio (RR) and its 95% confidence interval (CI) were determined using Stata statistical software version 14.0. The *p* values were two sided, with an alpha level of 0.05 considered significant. The heterogeneity across studies was quantified using the I2 statistic (0%–25% low heterogeneity, 25%–50% moderate heterogeneity, 50%–75% substantial heterogeneity, 75%–100% high heterogeneity).

To identify potential sources of subgroup differences and observed heterogeneity, we performed subgroup analyses on median year of publication (before 2015 vs. after 2015), study population (United States vs. China), and duration of follow-up (long-term vs. short-term). To evaluate the robustness of pooled results, we performed sensitivity analyses by excluding studies one by one. Potential publication bias was assessed by visualisation of asymmetry in funnel plots in combination with both Egger’s test and Begg’s test.

## 3 Result

**FIGURE 1:**
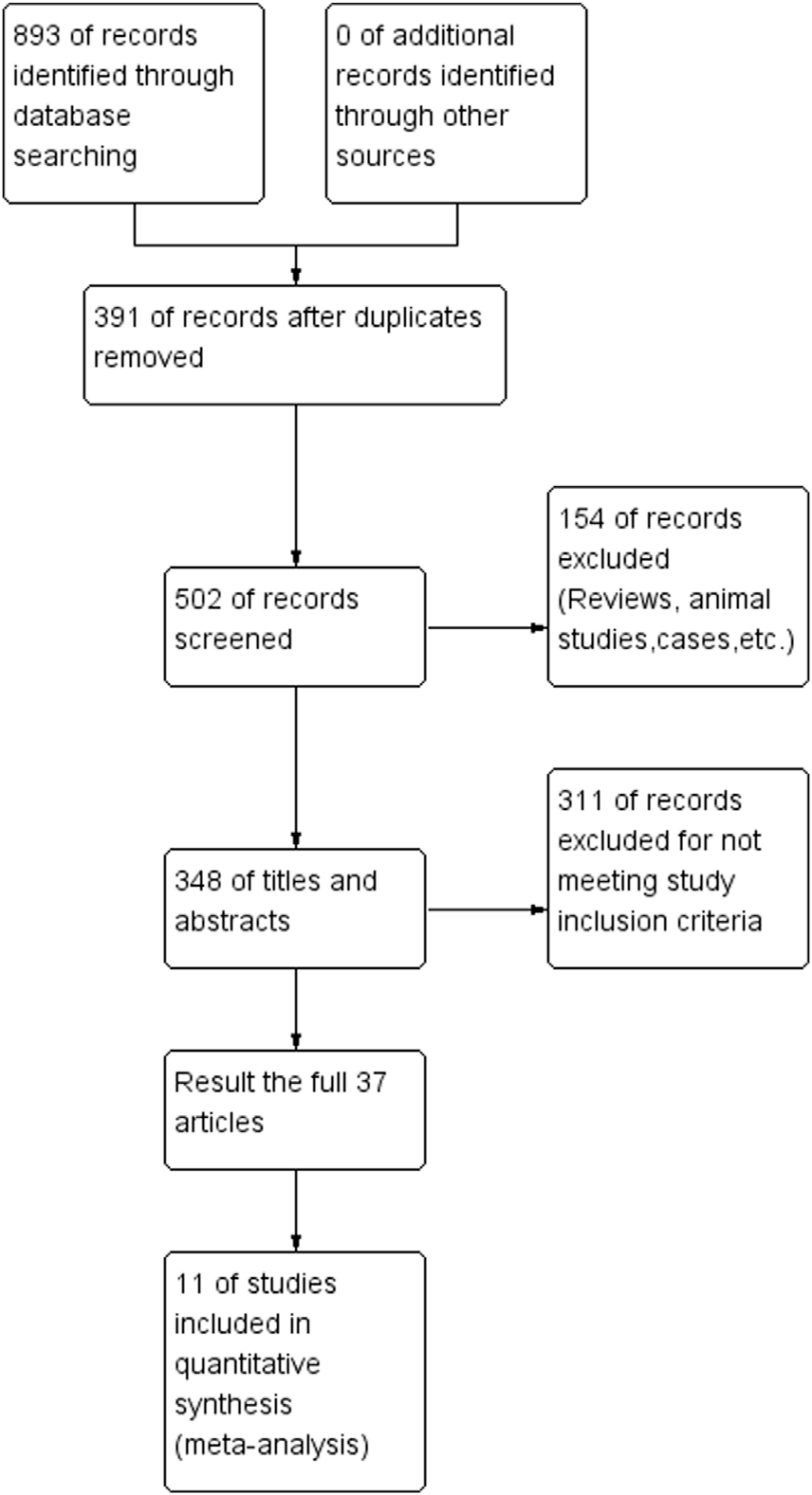
Flow chart of drugs in combination with stents versus drugs alone in the treatment of patients with sICAS.

A total of 893 studies were obtained from the initial search. After excluding duplicate literature, 502 remained. We conducted title/abstract screening and full text reading, resulting in 11 studies (Chimowitz et al., 2011; Zhu, 2011; Miao et al., 2012; Gao and Gao, 2013; Li, 2013; Derdeyn et al., 2014; Zaidat et al., 2015; Ma, 2018; Ding et al., 2021; Gao et al., 2022; Huang et al., 2022) being included in this meta-analysis. The search process is shown in Figure 1. A total of 1915 patients (951 in the stent group and 964 in the drug-only group) with a diagnosis of sICAS were included in this study.

### 3.1 Characteristics of included studies

The basic information of the included studies is detailed in Table 1. Three of the eleven studies were from the United States and 8 from China. Antiplatelet drug doses were mostly Aspirin 100 mg daily (Long-term use) and Clopidogrel 75 mg daily (90 days). Wingspan stent system was used for most of the US stents, and the majority of stenoses were greater than or equal to 70%. Eleven studies used the rate of transient ischaemia or stroke, any stroke or mortality, cerebral haemorrhage, disabling stroke or death as the primary outcome. Six studies (Zhu, 2011; Miao et al., 2012; Li, 2013; Zaidat et al., 2015; Ma, 2018; Huang et al., 2022) used the rate of TIA or stroke as the primary outcome for 9 effect sizes. Six studies (Chimowitz et al., 2011; Zhu, 2011; Gao and Gao, 2013; Derdeyn et al., 2014; Ding et al., 2021; Gao et al., 2022)used the rate of any stroke or death as the primary outcome for 9 effect sizes. Three studies (Chimowitz et al., 2011; Derdeyn et al., 2014; Zaidat et al., 2015) had cerebral haemorrhage as the primary outcome for 4 effect measures. Five studies (Chimowitz et al., 2011; Miao et al., 2012; Derdeyn et al., 2014; Zaidat et al., 2015; Gao et al., 2022) had either a disabling stroke rate or a death rate as the primary outcome for 6 effect measures.

**FIGURE 2:**
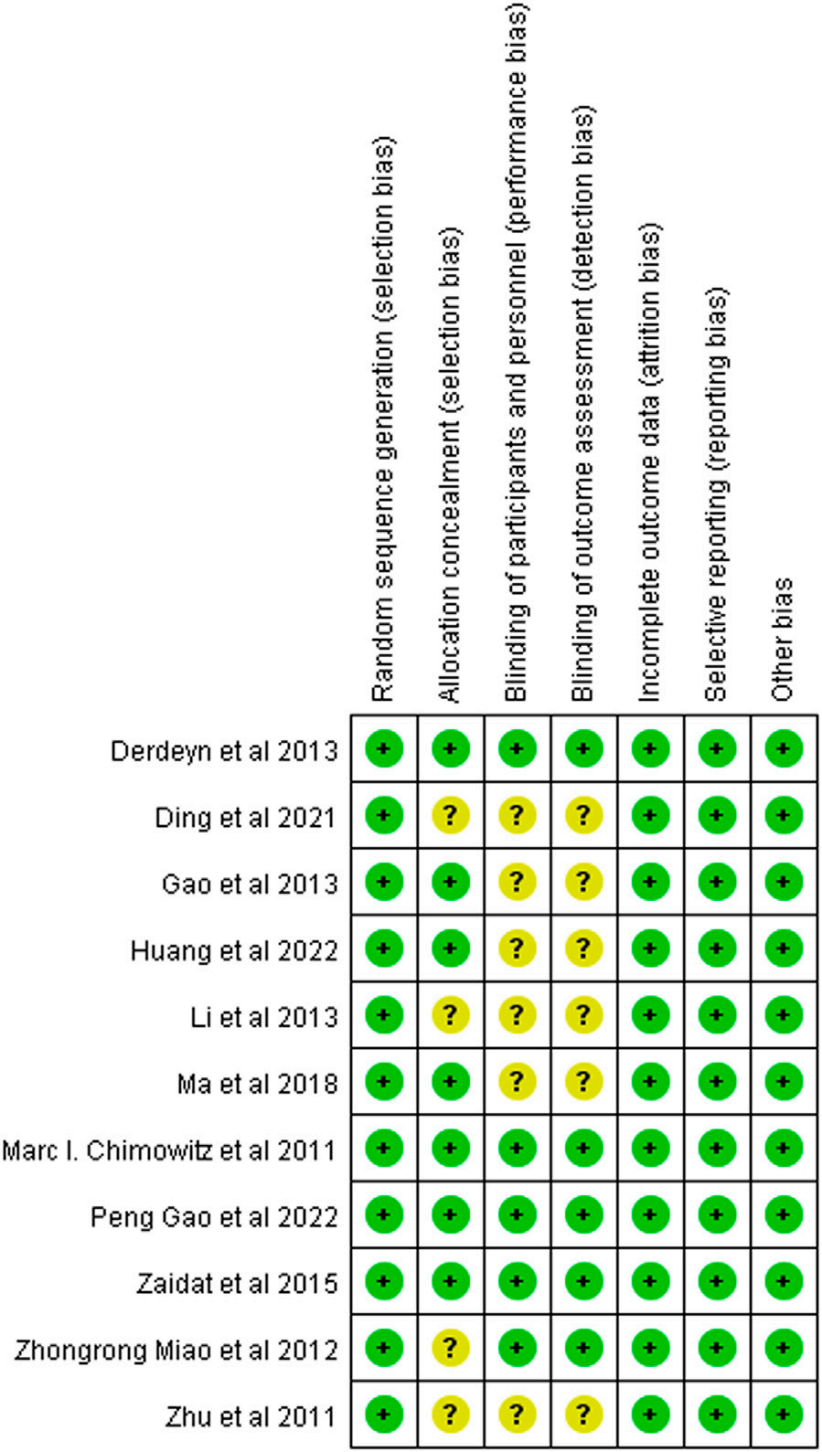
Summary chart of the risk of bias in patients with sICAS treated with drugs in combination with stents versus drugs alone.

The Chinese literature was low in terms of blinded scores, but the Jadad scale scores were all >3, so all included studies were of high quality (Figure 2).

### 3.2 Effect of interventions

Details of outcomes of included studies are shown in Table 2.

**TABLE 2:** Results of a subgroup analysis of the safety and efficacy of two treatment modalities in patients with symptomatic intracranial artery stenosis.

Study characteristics	No. study	Risk ratio (95%CI)	I^2^ (%)	P for between groups	Subgroup		No. study	Risk ratio (95%CI)	I^2^ (%)	P for within groups
Stroke or TIA	9	0.65 (0.29,1.44)	71.8	0.286	Country	United States	2	2.11 (1.17,3.80)	0	0.013^a^
						China	7	0.41 (0.17,1.00)	71.8	0.051
					Year of publication	before 2015	5	0.44 (0.12,1.59)	72.2	0.211
						after 2015	4	0.94 (0.33,2.72)	73.4	0.911
					Follow-up time	Long-term	7	0.46 (0.17,1.22)	75.2	0.103
						Short-term	2	2.01 (0.87,4.66)	0	0.119
Stroke or death	9	1.33 (1.06,1.67)	42.8	0.015	Country	United States	3	1.49 (1.16,1.91)	31	0.002^a^
						China	6	0.77 (0.43,1.38)	36.3	0.382
					Year of publication	before 2015	6	1.39 (1.09,1.78)	26.9	0.008^a^
						after 2015	3	0.99 (0.52,1.89)	66.2	0.977
					Follow-up time	Long-term	6	1.29 (1.02,1.63)	58.1	0.033^a^
						Short-term	3	2.03 (0.77,5.39)	0	0.154
Cerebral haemorrhage	4	12.63 (3.93,40.58)	0	<0.0001	Follow-up time	Long-term	2	11.59 (2.76,48.77)	0	0.001^a^
						Short-term	2	14.64 (1.98,108.25)	0	0.009^a^
Disabling stroke or death	6	1.512 (1.089,2.099)	0	0.013	Country	United States	4	1.61 (1.09,2.36)	5.5	0.016^a^
						China	2	1.28 (0.68,2.40)	0	0.448
					Year of publication	before 2015	4	1.58 (1.06,2.36)	3.2	0.025^a^
						after 2015	2	1.38 (0.78,2.44)	0	0.271
					Follow-up time	Long-term	4	1.33 (0.94,1.89)	0	0.110
						Short-term	2	3.53 (1.26,9.86)	0	0.016^a^

^a^ Indicates *p* < 0.05; Transient cerebral ischaemia = TIA; Long-term = Outcome indicators at 1, 2 or 3 years of follow-up,Short-term = Outcome indicators for follow-up ≤ 30 days.

#### 3.2.1 Stroke or TIA

**FIGURE 3:**
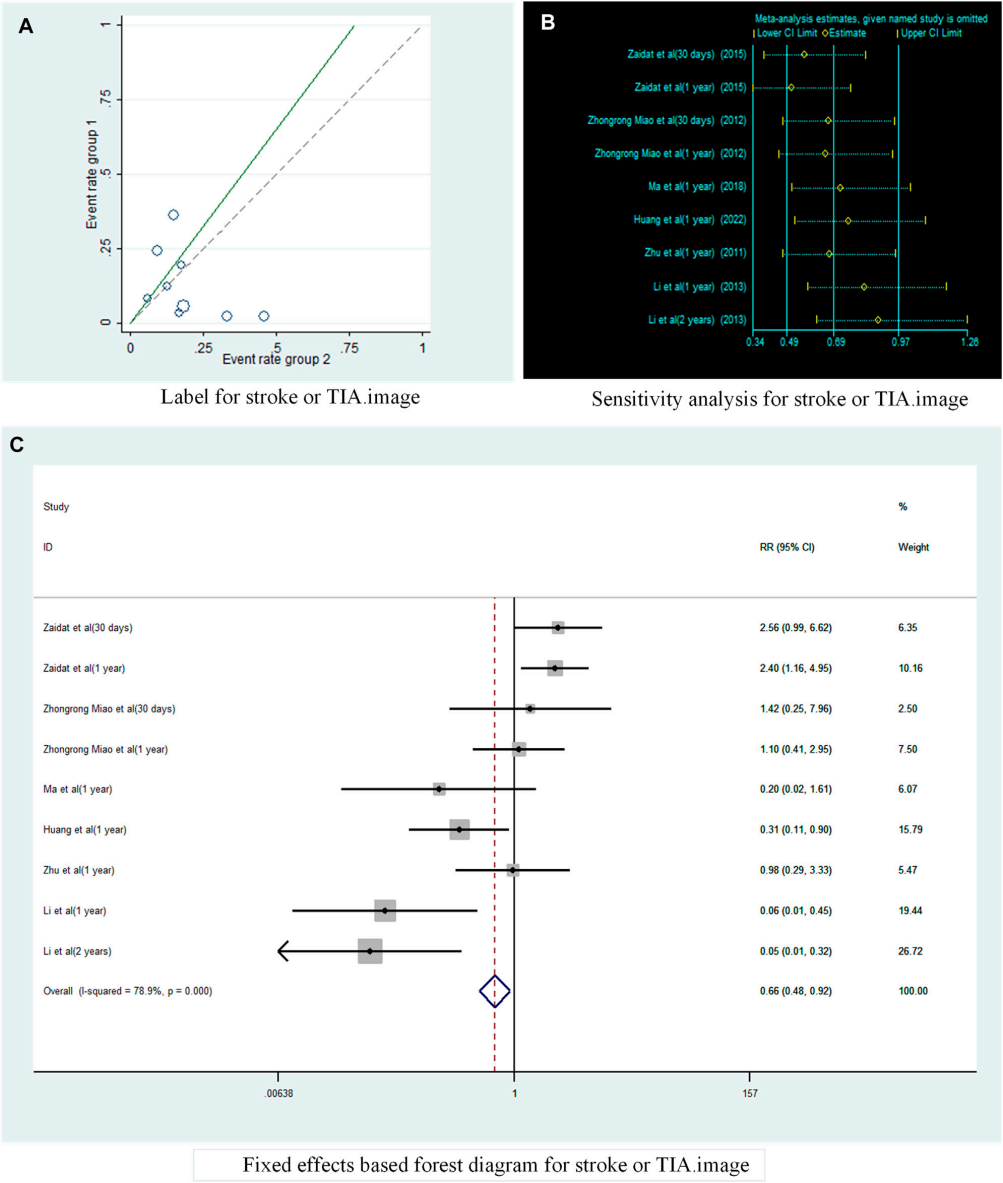
Analysis of heterogeneity in the occurrence of Stroke or TIA in patients with sICAS treated with drugs in combination with stents versus drugs alone. **(A)** Label for stroke or TIA. **(B)** Sensitivity analysis for stroke or TIA. **(C)** Fixed effects based on forest diagram for stroke or TIA.

**FIGURE 4:**
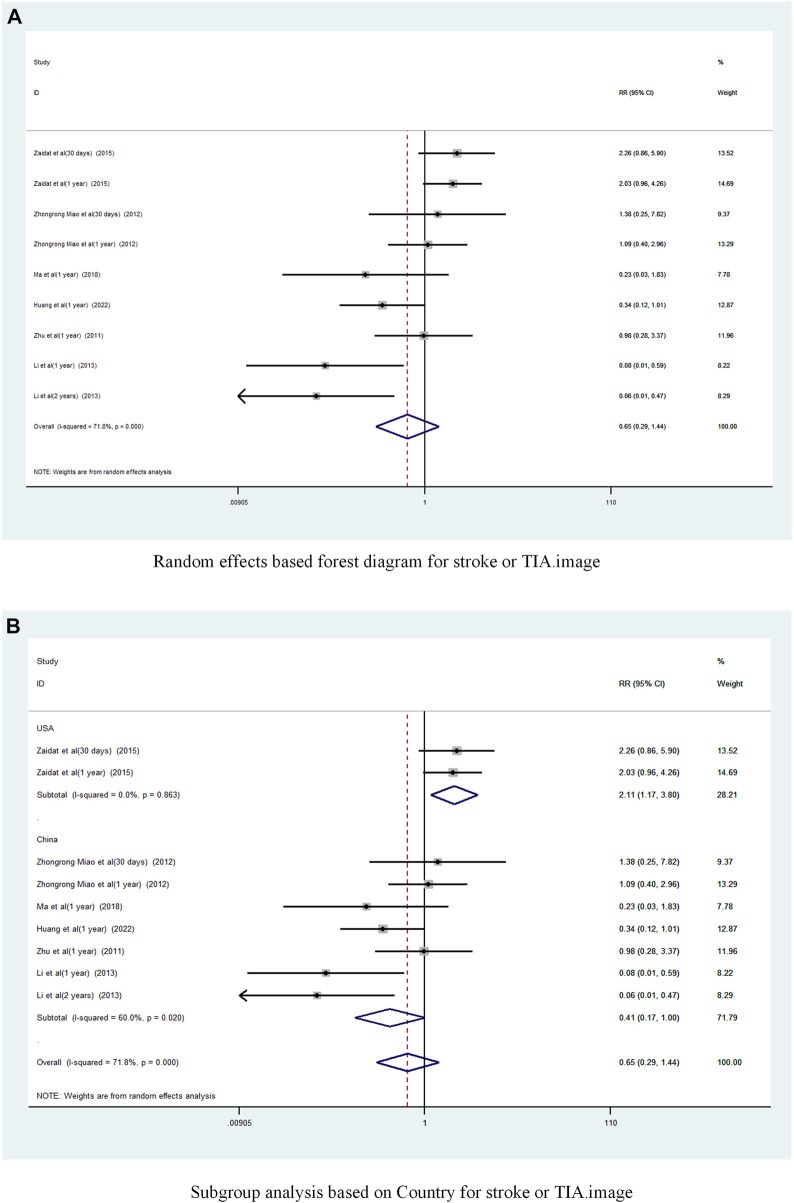
Forest plot of the occurrence of Stroke or TIA in patients with sICAS treated with drugs in combination with stents versus drugs alone. **(A)** Random effects based forest diagram for stroke or TIA. **(B)** Subgroup analysis based on Country for stroke or TIA.

A total of six studies (Zhu, 2011; Miao et al., 2012; Li, 2013; Zaidat et al., 2015; Ma, 2018; Huang et al., 2022) reported the incidence of TIA or stroke in patients with sICAS treated with stents combined with drugs compared to drugs alone. Label (Figure 3A) and forest plot (Figure 3C) showed a high degree of differentiation between studies (I^2^ = 78.9%, *p* < 0.001). The analysis showed that in six studies with a pooled RR of 0.65 (95% CI 0.29 to 1.44, I^2^ = 71.8%) (Figure 4A), there was no significant difference in the incidence of transient ischaemia and stroke in patients with sICAS treated with stents in combination with drugs versus drugs alone.

Subgroup analysis by country (Figure 4B) showed that the consolidated risk ratio of the US Asian group is 2.11 (95% CI 1.17 to 3.80, I^2^ = 0%) and the consolidated risk ratio of the Chinese Asian group is 0.41 (95% CI 0.17 to 1.00, I^2^ = 71.8%). The USA subgroup analysis showed that stenting combined with drug therapy significantly increased the incidence of TIA and stroke in patients compared with drug therapy alone.

**FIGURE 5:**
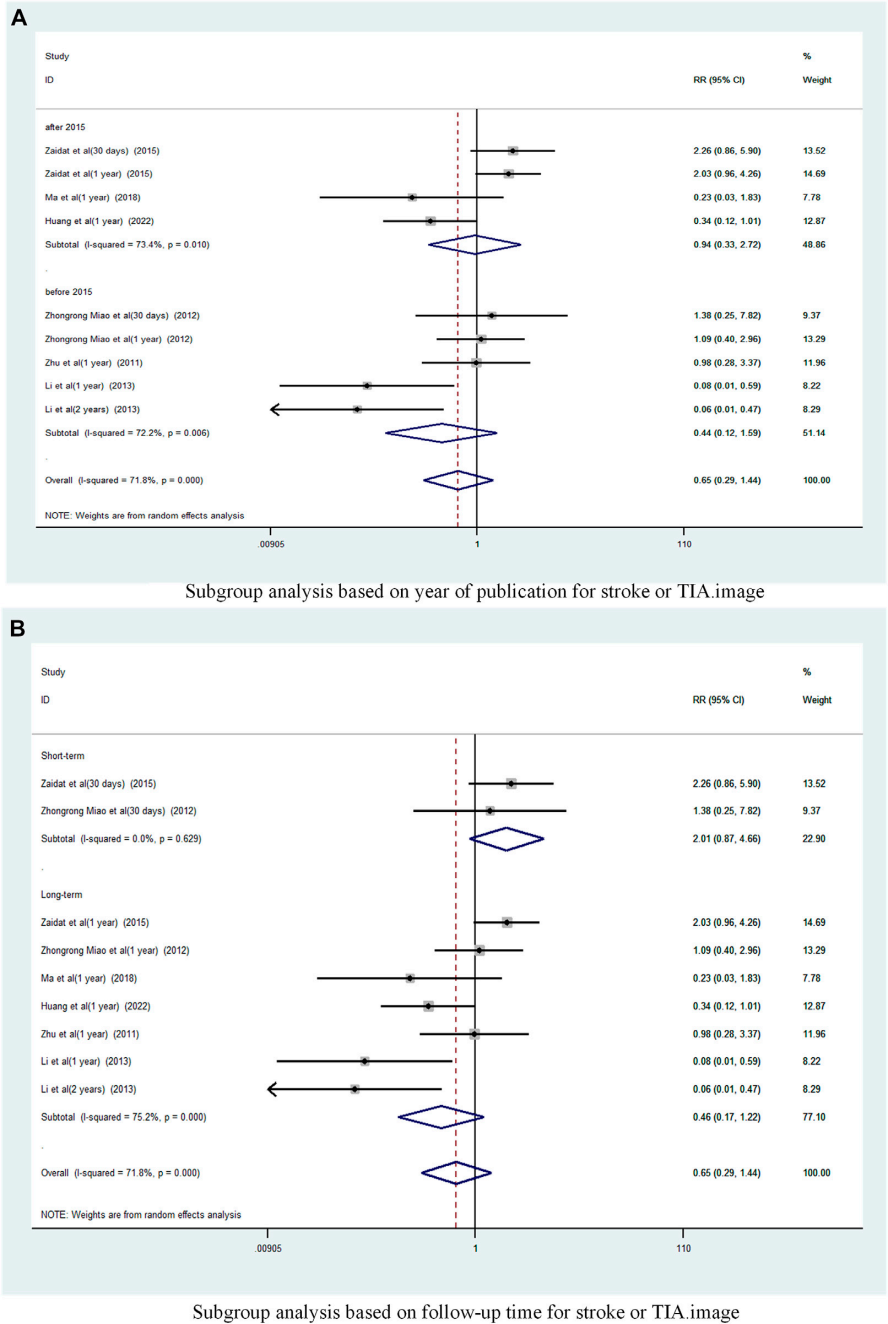
Subgroup analysis of the incidence of stroke or TIA in patients with sICAS treated with drugs combined with stenting versus drugs alone. **(A)** Subgroup analysis based on year of publication for stroke or TIA. **(B)** Subgroup analysis based on follow-up time for stroke or TIA.

A subgroup analysis based on Year of publication showed (Figure 5A), the risk ratio of the consolidated risk before 2015 is 0.44 (95% CI 0.12 to 1.59, I^2^ = 72.2%), the risk ratio of the consolidation risk after 2015 is 0.94 (95% CI 0.33 to 2.72, I^2^ = 73.4%). There was no significant difference in the incidence of TIA and stroke in patients treated with the stent-drug combination compared with drug therapy alone, either before or after 2015.

Subgroup analysis based on follow-up time (Figure 5B) showed that Long-term with pooled risk ratios of 0.46 (95% CI 0.17 to 1.22, I^2^ = 75.2%), Short-term with pooled risk ratios of 2.01 (95% CI 0.87 to 4.66, I^2^ = 0%). There was no significant difference in the incidence of TIA and stroke between patients treated with the combination of drugs in the Long-term and Short-term stents compared to those treated with drugs alone.

**FIGURE 6:**
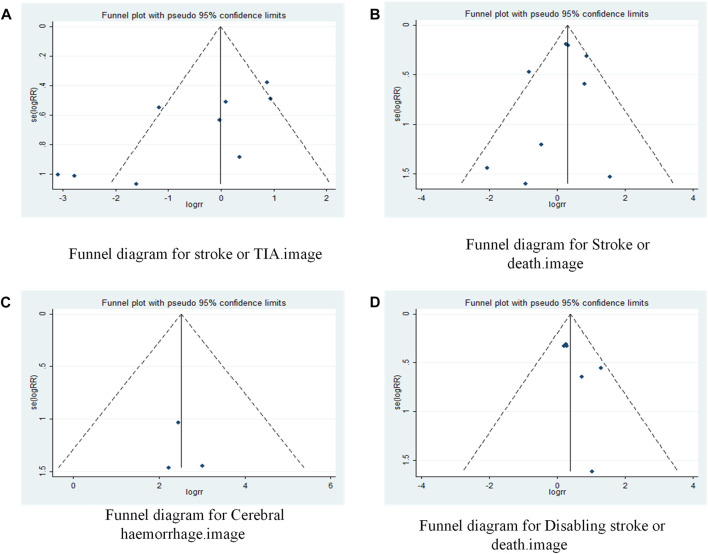
Funnel plot of drugs in combination with stents versus drugs alone in the treatment of patients with sICAS. **(A)** Funnel diagram for stroke or TIA. **(B)** Funnel diagram for Stroke or death. **(C)** Funnel diagram for Cerebral haemorrhage. **(D)** Funnel diagram for Disabling stroke or death.

A funnel plot was drawn to test for the risk of publication bias (Figure 6A). *p* = 048 < 0.05 for Begg’s test, indicating that there may be some publication bias in the included literature.

#### 3.2.2 Stroke or death

**FIGURE 7:**
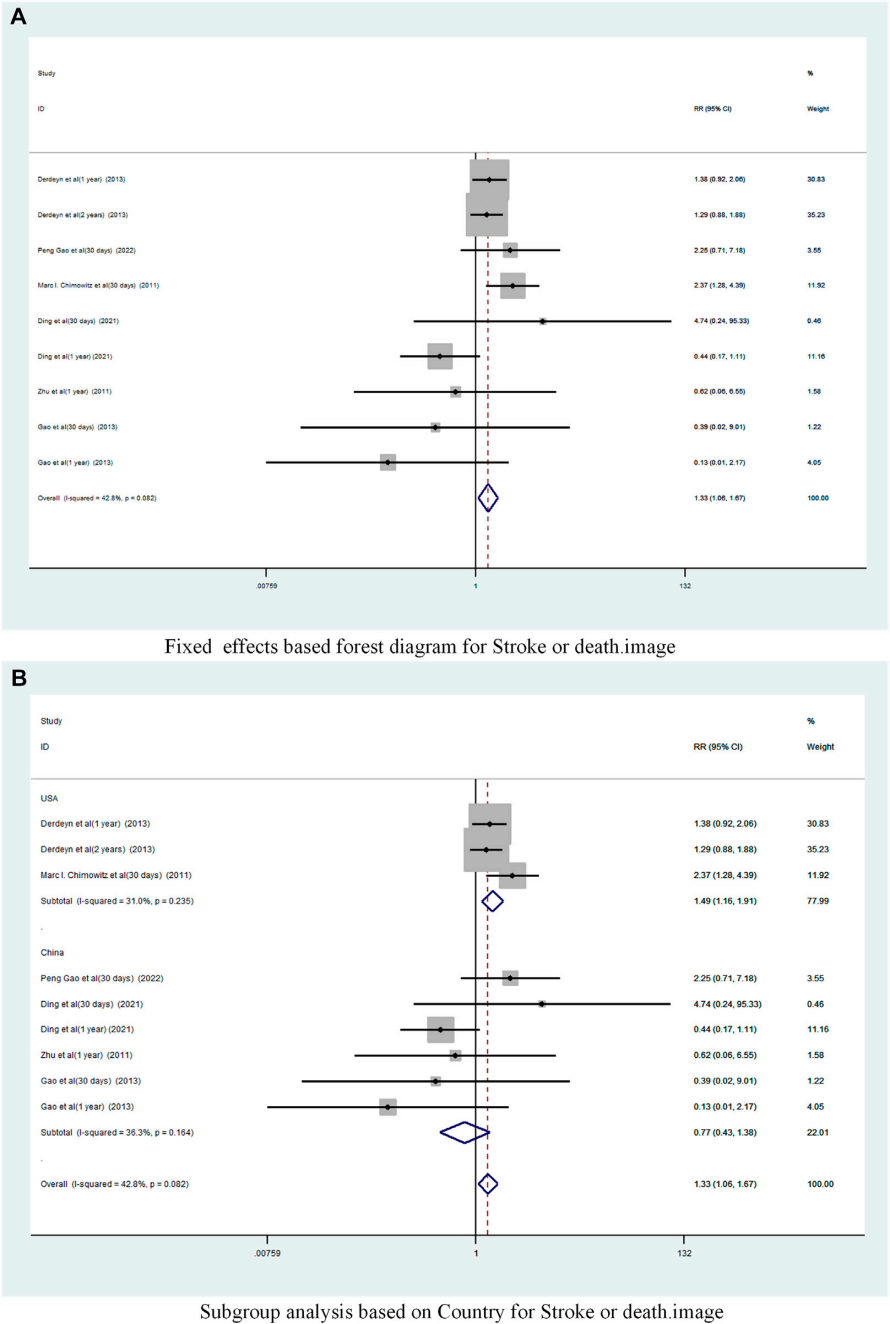
Forest plot of the occurrence of Stroke or death in patients with sICAS treated with drugs in combination with stents versus drugs alone. **(A)** Fixed effects based forest diagram for Stroke or death. **(B)** Subgroup analysis based on Country for Stroke or death.

A total of 6 studies (Chimowitz et al., 2011; Zhu, 2011; Gao and Gao, 2013; Derdeyn et al., 2014; Ding et al., 2021; Gao et al., 2022) reported on the incidence of Stroke or death in patients with sICAS treated with stents in combination with drugs compared to drugs alone. Forest plots (Figure 7A) showed a low degree of heterogeneity between studies (I^2^ = 42.8%, *p* = 0.082), and effect sizes were combined using a fixed effects model. The analysis showed a pooled RR of 1.33 (95% CI 1.06 to 1.67, *p* = 0.015) across the six studies (Figure 7A) and a significantly increased incidence of death and stroke in patients with sICAS treated with stenting combined with medication.

Subgroup analysis by country (Figure 7B) showed that the consolidated risk ratio of the US Asian group is 1.49 (95% CI 1.16 to 1.91, I^2^ = 31.0%) andthe consolidated risk ratio of the Chinese Asian group is 0.77 (95% CI 0.43 to 1.38, I^2^ = 36.3%). The USA subgroup analysis showed that stenting combined with medication significantly increased the incidence of Stroke or death in patients compared to medication alone.

**FIGURE 8:**
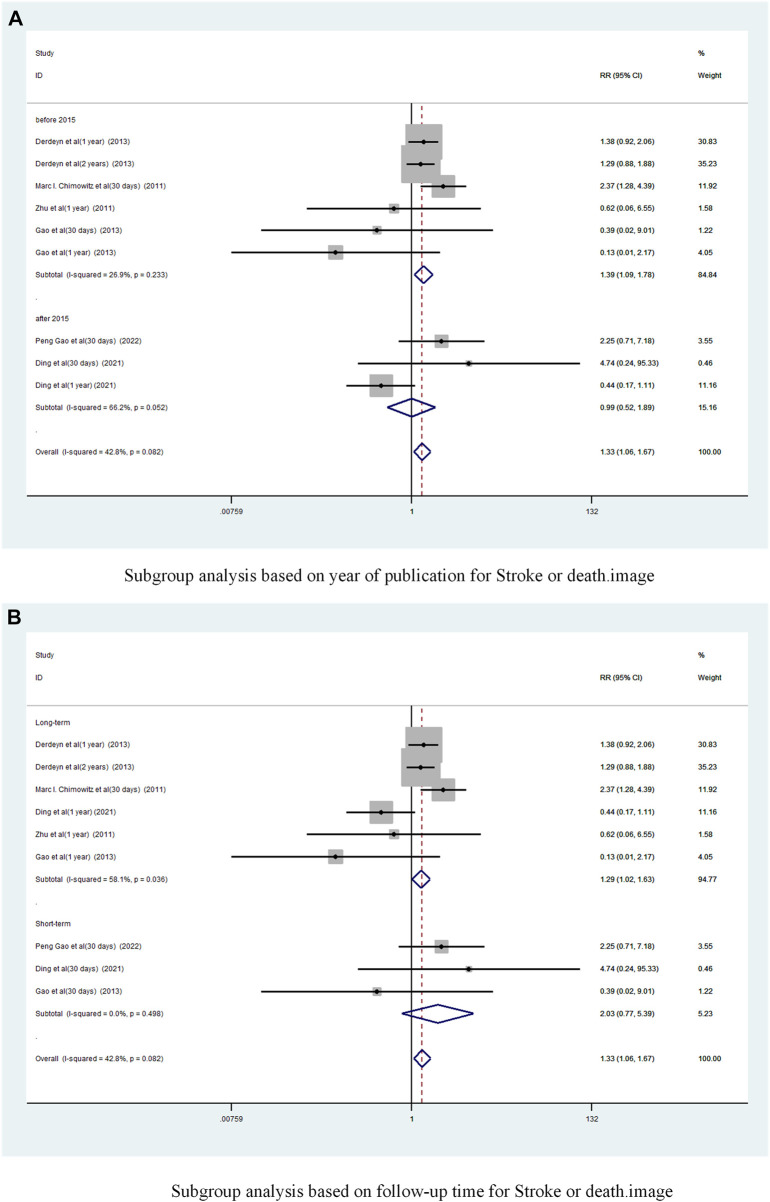
Subgroup analysis of the incidence of stroke or death in patients with sICAS treated with drugs combined with stenting versus drugs alone. **(A)** Subgroup analysis based on year of publication for Stroke or death. **(B)** Subgroup analysis based on follow-up time for Stroke or death.

Subgroup analysis based on year of publication (Figure 8A) showed a pooled RR of 1.39 (95% CI 1.09 to 1.78, I^2^ = 26. 9%) before 2015 and 0.99 (95% CI 0.52 to 1.89, I^2^ = 66.2%) after 2015. Befor 2015 stent combination drugs significantly increased the occurrence of stroke or death in patients.

Subgroup analysis based on follow-up time showed (Figure 8B) that the long-term versus pooled RR was 1.29 (95% CI 1.02 to 1.63, I^2^ = 58. 1%) and the short-term pooled RR was 2.03 (95% CI 0.77 to 5.39, I^2^ = 0%). Compared to less than 30 days, stenting combined with drugs significantly increased the incidence of stroke or death at greater than 1 year.

A funnel plot was drawn to test for the risk of publication bias (Figure 6B). *p* = 0.602 > 0.05 for Begg’s test, indicating that the likelihood of publication bias in the included literature was relatively small.

#### 3.2.3 Cerebral haemorrhage

**FIGURE 9:**
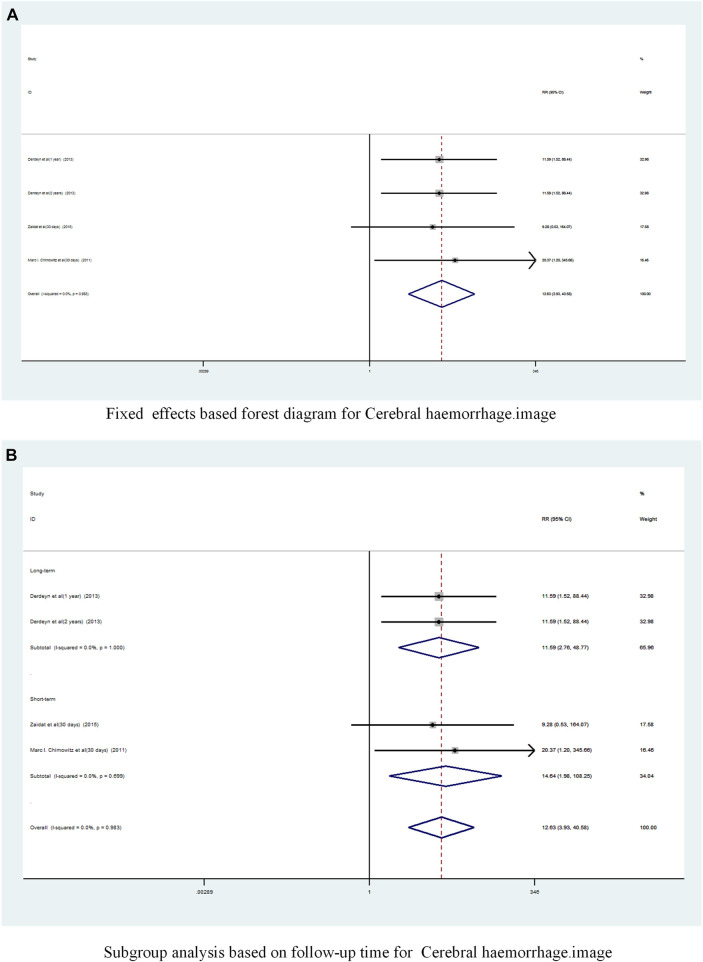
Forest plot of the occurrence of Cerebral haemorrhage in patients with sICAS treated with drugs in combination with stents versus drugs alone. **(A)** Fixed effects based forest diagram for Cerebral haemorrhage. **(B)** Subgroup analysis based on follow-up time for Cerebral haemorrhage.

A total of 3 studies (9,10,13])reported on the incidence of Cerebral haemorrhage in patients with sICAS treated with stents in combination with drugs compared to drugs alone. Forest plots (Figure 9A) showed a low degree of heterogeneity between studies (I^2^ = 0%, *p* = 0.983), and effect sizes were combined using a fixed effects model. Analysis showed that 3 studies, with pooled risk ratios of 12.63 (95% CI 3.93 to 40.58, *p* < 0.0001) (Figure 9A), stent combined with medication significantly increased the probability of Cerebral haemorrhage in patients with sICAS.

A subgroup analysis based on follow-up time showed (Figure 9B) that the long-term pooled RR was 11.59 (95% CI 2.76 to 48.77, I^2^ = 0%) and the short-term pooled RR was 14. 64 (95% CI 1.98 to 108.25, I^2^ = 0%). Stenting combined with drugs significantly increased the incidence of cerebral haemorrhage in patients at both long-term and short-term follow-up.

A funnel plot was drawn to test for the risk of publication bias (Figure 6C). *p* = 0.172 > 0.05 for Egger’s test, indicating that the likelihood of publication bias in the included literature was relatively small.

#### 3.2.4 Disabling stroke or death

**FIGURE 10:**
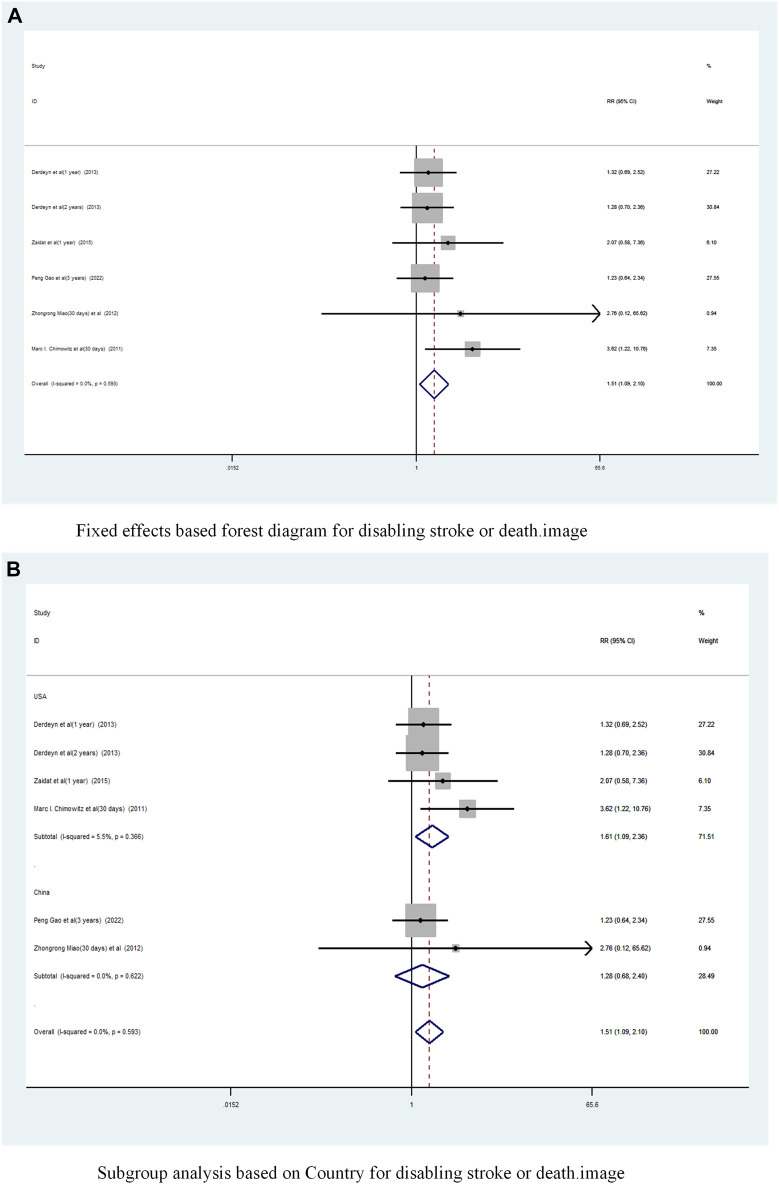
Forest plot of disabling stroke or death in patients with sICAS treated with drugs in combination with stents versus drugs alone. **(A)** Fixed effects based forest diagram for disabling stroke or death. **(B)** Subgroup analysis based on Country for disabling stroke or death.

A total of 5 studies (Chimowitz et al., 2011; Miao et al., 2012; Derdeyn et al., 2014; Zaidat et al., 2015; Gao et al., 2022) reported on the incidence of Disabling stroke or death in patients with sICAS treated with stents combined with drugs compared to drugs alone. Forest plots (Figure 10A) showed a low degree of heterogeneity between studies (I^2^ = 0%, *p* = 0.593), and effect sizes were combined using a fixed effects model. The analysis showed that in 5 studies, with pooled risk ratios of 1.51 (95% CI 1.089 to 2.10, *p* = 0.013) (Figure 10A), stent combination drugs significantly increased the probability of Disabling stroke or death in patients with sICAS.

Subgroup analysis by country (Figure 10B) showed that the consolidated risk ratio of the US Asian group is 1.61 (95% CI 1.09 to 2.36, I^2^ = 5.5%) and he consolidated risk ratio of the Chinese Asian group is 1.28 (95% CI 0.68 to 2.40, I^2^ = 0%). The USA subgroup analysis showed that stenting combined with medication significantly increased the incidence of Disabling stroke or death in patients compared to medication alone.

**FIGURE 11:**
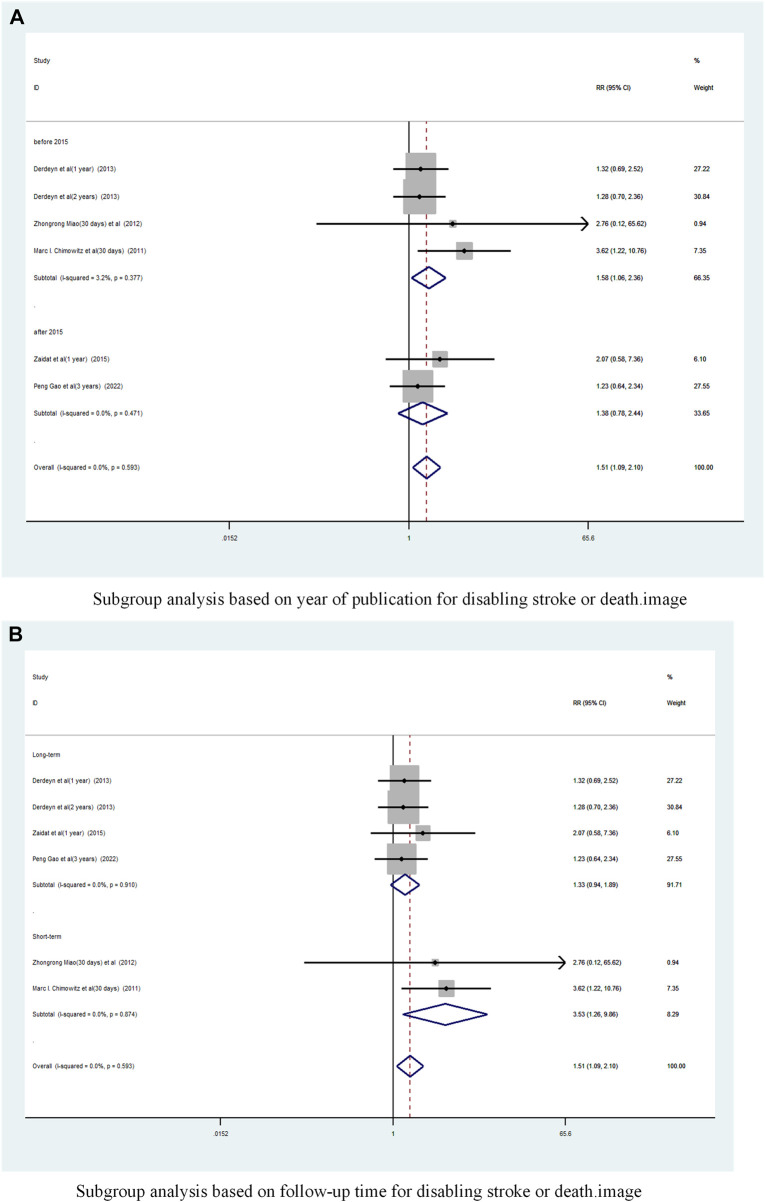
Subgroup Analysis of Disabling Stroke or Death in Patients with sICAS Treated with Drug Combination Stents Compared to Drugs Alone. **(A)** Subgroup analysis based on year of publication for disabling stroke or death. **(B)** Subgroup analysis based on follow-up time for disabling stroke or death.

A subgroup analysis based on Year of publication showed (Figure 11A), the risk ratio of the consolidated risk before 2015 is 1.58 (95% CI 1.059 to 2.358, I^2^ = 3.2%), the risk ratio of the consolidation risk after 2015 is 1.38 (95% CI 0.78 to 2.44, I^2^ = 0%). Stenting combined with drug treatment before 2015 significantly increased the probability of Disabling stroke or death in patients.

Subgroup analysis based on follow-up time showed (Figure 11B) that the pooled RR for long-term follow-up was 1.33 (95% CI 0.94 to 1.89, I^2^ = 0%) and for short-term was 3. 53 (95% CI 1.26 to 9.86, I^2^ = 0%). Short-term follow-up time stents combined with drugs significantly increased the probability of disabling stroke or death in patients.

A funnel plot was drawn to test for the risk of publication bias (Figure 6D). *p* = 0.260 > 0.05 for Begg’s test, indicating that the likelihood of publication bias in the included literature was relatively small.

### 3.3 Sensitivity analysis

**FIGURE 12:**
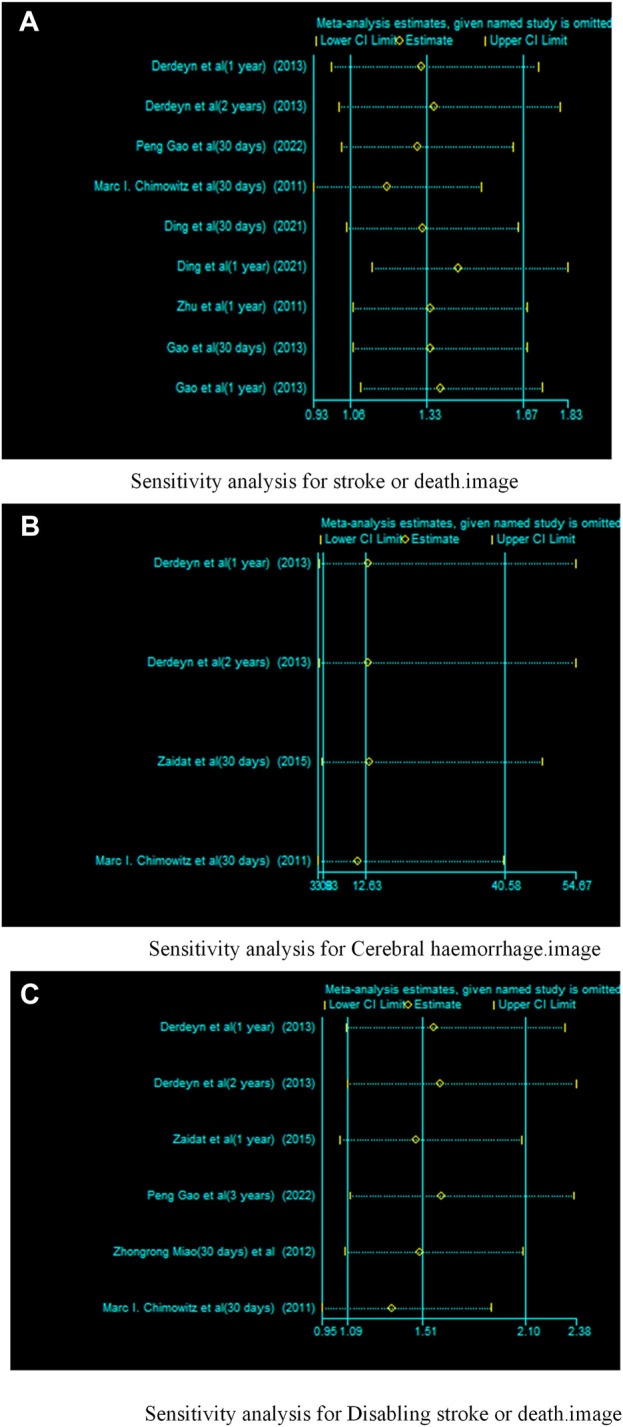
Sensitivity analysis of drugs in combination with stents versus drugs alone in the treatment of patients with sICAS. **(A)** Sensitivity analysis for stroke or death. **(B)** Sensitivity analysis for Cerebral haemorrhage. **(C)** Sensitivity analysis for disabling stroke or death.

We conducted sensitivity analyses for each outcome (Figure 12). Removing each study individually did not change the direction of the effect size for any of the results and the validation results were stable.

## 4 Discussion

Aggressive medical managemen is recommended as the first-line therapy for symptomatic sICAS by the American Heart Association stroke prevention guidelines (Kernan et al., 2014). Although the results of the Stenting with Aggressive Medical Therapy for Intracranial Artery Stenosis (SAMMPRIS) and Vitesse Intracranial Stenting for Ischemic Stroke Treatment (VISSIT) trials did not favour the use of senting in patients with sICAS, but many neurovascular practitioners and academics continue to believe that there is a role for endovascular treatment of sICAS(1). Recent multicentre clinical studies have shown no significant difference in the risk of stroke or death within 30 days between percutaneous fluoroscopic angioplasty and stenting compared with drug treatment alone in patients with symptoms of severe intracranial atherosclerotic stenosis, nor was there a significant difference in the risk of stroke in the region of the qualifying artery beyond 30 days at 1 year (Gao et al., 2022). The use of stenting for sICAS is technically feasible, but whether patients with severe stenosis are at higher risk of recurrent target lesion-associated ischaemic stroke after stenting, and in particular the near and long-term outcomes of stenting and its comparison with pharmacological treatment alone, remain unclear and lack further evidence to support this. Although there are some reports of stenting for sICAS, they are mostly single-centre, small-sample retrospective studies. We therefore conducted a systematic evaluation of RCTs of stenting for sICAS in an attempt to clarify the effectiveness and safety of stenting compared with drug-only treatment.

A total of 11 RCTs comparing the efficacy and safety of stent-combined drug therapy with drug therapy alone for sICAS were included in this pooled analysis by searching the literature. Our meta-analysis showed no significant difference in the incidence of TIA and stroke in patients with sICAS treated with stents in combination with drugs versus drugs alone. The incidence of death and stroke, cerebral haemorrhage, stroke or death were significantly increased in patients with sICAS treated with stents combined with drugs. According to a subgroup analysis conducted by country, the odds of stroke or TIA, stroke or death, and disabling stroke or death were significantly higher in the United States population using stents in combination with drugs for sICAS than drugs alone. According to a subgroup analysis conducted by year of publication, the before 2015 study showed that patients treated with stents in combination with drugs for sICAS were significantly more likely to experience stroke or death, disabling stroke or death than drugs alone. A subgroup analysis based on follow-up time showed that patients in the Long-term study using stents in combination with drugs for sICAS were significantly more likely to develop stroke or death, cerebral haemorrhage than drugs alone. A subgroup analysis based on follow-up time showed that patients in the short-term study using stents in combination with drugs for sICAS were significantly more likely to develop cerebral haemorrhage, disabling stroke or death than drugs alone. Subgroup difference tests showed statistically significant subgroup effects for country, year of publication, and follow-up time, suggesting that these factors may have statistically significantly altered the association between these two treatment modalities and recurrence rates.

The Wingspan (Henkes et al., 2005) study showed that the stent system is clinically effective in treating high-risk symptomatic patients who do not respond well to medication, and the FDA approved the stent system for use in people at high risk of recurrent cerebrovascular events. Endovascular stenting is a special umbrella to prevent dislodged blood clots from entering the intracranial vessels, allowing the narrowed and occluded vessels to dilate and recanalize, conforming to the direction of medical development with significant efficacy, minimal trauma and lower risk (Markaki et al., 2013). Previous studies on ischemic Stroke have shown different rates of mortality, probably owing to heterogeneous groups of patients (Kolominsky-Rabas et al., 2001). The results of this pooled analysis based on subgroup analysis of the study countries are likely to be influenced by population heterogeneity. Studies from Asia have reported lower rates of symptomatic ISR, consistent with the results of this study (Yu et al., 2014). It has been shown that elevated bilirubin levels are associated with more severe stroke severity (Pineda et al., 2008). In a population-based survey, higher serum total bilirubin levels were associated with lower stroke incidence and improved functional outcome (Perlstein et al., 2008). A study including patients with sICAS treated with balloon angioplasty showed a significantly higher event rate in patients with lengths greater than 10 mm than in other lesion types. However, other recent studies, using intracranial stents as primary treatment, did not find a significant correlation between lesion length and risk of perioperative complications (Suh et al., 2008; Kurre et al., 2010; Qureshi et al., 2011). Furthermore, it is possible that the event rates in SAMMPRIS do not reflect current event rates with stenting due to increased operator experience with stenting in general. In fact, the 30-day complication rates in the WEAVE trial were much lower than in the SAMMPRIS trial. It is very difficult to draw conclusions on the reasons for this, however (Alexander et al., 2019). While operator experience has been suggested, in the SAMMPRIS trial there were no significant differences in event rates among operators with varying degrees of experience with the Wingspan system (Derdeyn et al., 2013). Therefore, it is important that analyses are performed not only for all cases of vertebral stenosis, but also stratified by location. Further differences potentially affecting the trials’ results include the use of different stenting devices, and different drug treatment regimens in the stenting and medical treatment arms. Recent data emphasize that, whether the patient undergoes stenting or not, intensive medical therapy is important. This includes antiplatelet medications, risk factor management, and lifestyle measures (Drazyk and Markus, 2018). A recent meta-analysis (Wong et al., 2013) of studies has shown that dual therapy is more effective than monotherapy in reducing the risk of early recurrence of stroke in patients with acute stroke or TIA. We propose that future clinical trials investigating the benefit of stenting in severe ssICAS be based on identifying and selecting a subgroup of patients who are likely to fail aggressive medical treatment and whose risk on medical treatment is higher than the peri-procedural risk of stenting (Yaghi et al., 2020).

## 5 Limitations

In the heterogeneity test, we found heterogeneity in some outcomes. This was particularly true for the incidence of Stroke or TIA. Considering the interference of multiple factors such as country of study population, year of publication, length of follow-up, type of stent, degree of stenosis, diseased vessel, and dose of antiplatelet drugs used, we speculate that the sources of high heterogeneity may be very complex. Based on the data from the study, we were unable to complete an analysis of all sources of heterogeneity. It had some publication bias in the incidence results for Stroke or TIA, but the Begg’s test of *p* = 0.048 was very close to the critical value. The sensitivity analysis results were all relatively stable. Overall, the quality ratings for these studies were relatively reliable. Some of the studies we included had unclear randomisation and blinding treatments, which reduced the quality of the literature, but all of the literature quality assessment scores were greater than 4 and were of high quality. Six of the studies were single-centre and had a high failure to defend rate, thus having an impact on the strength of evidence for the effectiveness and safety evaluations in this pooled analysis. In addition, differences in operator experience with the procedure, site of sICAS, degree of stenosis, drug treatment dose and stent type were not standardised and may also have had some impact on the analysis of the results. Future trials of RCTs should take care to use blinding as far as possible, describe the randomisation method in detail and enhance the training of the surgical skills of the doctors included in the study, and conduct multicentre trials with large samples.

## 6 Conclusion

Studies suggest that stenting combined with medication for patients with sICAS may increase the incidence of death or stroke, cerebral haemorrhage, stroke or death, but has no significant effect on the incidence of TIA and stroke. The studies report inadequate and conflicting data and therefore the safety and efficacy of stenting for sICAS should be interpreted with caution. In the current situation, stenting is still not recommended as an initial treatment for patients with sICAS. More scientific conclusions need to be validated by a large amount of multicentre clinical data.

## Data Availability

The original contributions presented in the study are included in the article/Supplementary Materials, further inquiries can be directed to the corresponding author.
