# Supplementary material for: Safety and efficacy of stenting for symptomatic intracranial artery stenosis: a systematic reveiw and meta-analysis
Source: Front Pharmacol. 2023 Jun 8;14:1122842. doi: 10.3389/fphar.2023.1122842 (PMC10285394; doi:10.3389/fphar.2023.1122842)

Safety and efficacy of stenting for symptomatic intracranial artery stenosis: a systematic reveiw and meta-analysis

Ting Shi, ShiJian Chen, YongPei Long,ZhongDeng Gu^∗^

∗ Correspondence: Zhong-Deng Gu:125314506@qq.com

Search in English：

1. patient:①：Mesh:Cerebral Infarctions

free：Infarctions, CerebralInfarction, Cerebral,Cerebral Infarct,Cerebral Infarcts,Infarct, Cerebral,Infarcts, Cerebral,Cerebral Infarction, Left Hemisphere,Left Hemisphere, Cerebral,Infarction,Infarction, Cerebral, Left Hemisphere,Cerebral, Left Hemisphere, InfarctionInfarction, Left Hemisphere, Cerebral,Left Hemisphere, Infarction, Cerebral,Subcortical Infarction,Infarction, Subcortical,Infarctions, Subcortical,Subcortical Infarctions,Posterior Choroidal Artery Infarction,Anterior Choroidal Artery Infarction,Cerebral Infarction, Right Hemisphere,Right Hemisphere, Cerebral Infarction,Infarction, Right Hemisphere, Cerebral,Right Hemisphere, Infarction, Cerebral,Cerebral, Right Hemisphere, Infarction,Infarction, Cerebral, Right Hemisphere②Intracranial atherosclerosis③Intracranial artery stenosis

2. Intervention: stenting

3. Control: drug treatment alone

4. study method: randomised controlled (randomised, RCT)

Pubmed Search Strategy：

Search: ((((("Cerebral Infarction"[Mesh]) OR ((((((((((((((((((((((((Infarctions, Cerebral[Title/Abstract]) OR (Infarction, Cerebral[Title/Abstract])) OR (Cerebral Infarct[Title/Abstract])) OR (Cerebral Infarcts[Title/Abstract])) OR (Infarct, Cerebral[Title/Abstract])) OR (Infarcts, Cerebral[Title/Abstract])) OR (Cerebral Infarction, Left Hemisphere[Title/Abstract])) OR (Left Hemisphere, Cerebral Infarction[Title/Abstract])) OR (Infarction, Cerebral, Left Hemisphere[Title/Abstract])) OR (Cerebral, Left Hemisphere, Infarction[Title/Abstract])) OR (Infarction, Left Hemisphere, Cerebral[Title/Abstract])) OR (Left Hemisphere, Infarction, Cerebral[Title/Abstract])) OR (Subcortical Infarction[Title/Abstract])) OR (Infarction, Subcortical[Title/Abstract])) OR (Infarctions, Subcortical[Title/Abstract])) OR (Subcortical Infarctions[Title/Abstract])) OR (Posterior Choroidal Artery Infarction[Title/Abstract])) OR (Anterior Choroidal Artery Infarction[Title/Abstract])) OR (Cerebral Infarction, Right Hemisphere[Title/Abstract])) OR (Right Hemisphere, Cerebral Infarction[Title/Abstract])) OR (Infarction, Right Hemisphere, Cerebral[Title/Abstract])) OR (Right Hemisphere, Infarction, Cerebral[Title/Abstract])) OR (Cerebral, Right Hemisphere, Infarction[Title/Abstract])) OR (Infarction, Cerebral, Right Hemisphere[Title/Abstract]))) OR (Intracranial artery stenosis)) OR (Intracranial atherosclerosis)) AND (("Stents"[Mesh]) OR (sent[Title/Abstract]))) AND (randomized controlled trial[Publication Type] OR (randomized[Title/Abstract] AND controlled[Title/Abstract] AND trial[Title/Abstract]))

Chinese search strategy.

1. disease type:subject term: cerebral infarction

Free word: intracranial artery stenosis, intracranial atherosclerosis

2. intervention: stent placement

3. control: drug therapy alone

4. study method: randomized controlled (randomized, RCT)

CNKI Search Strategy：


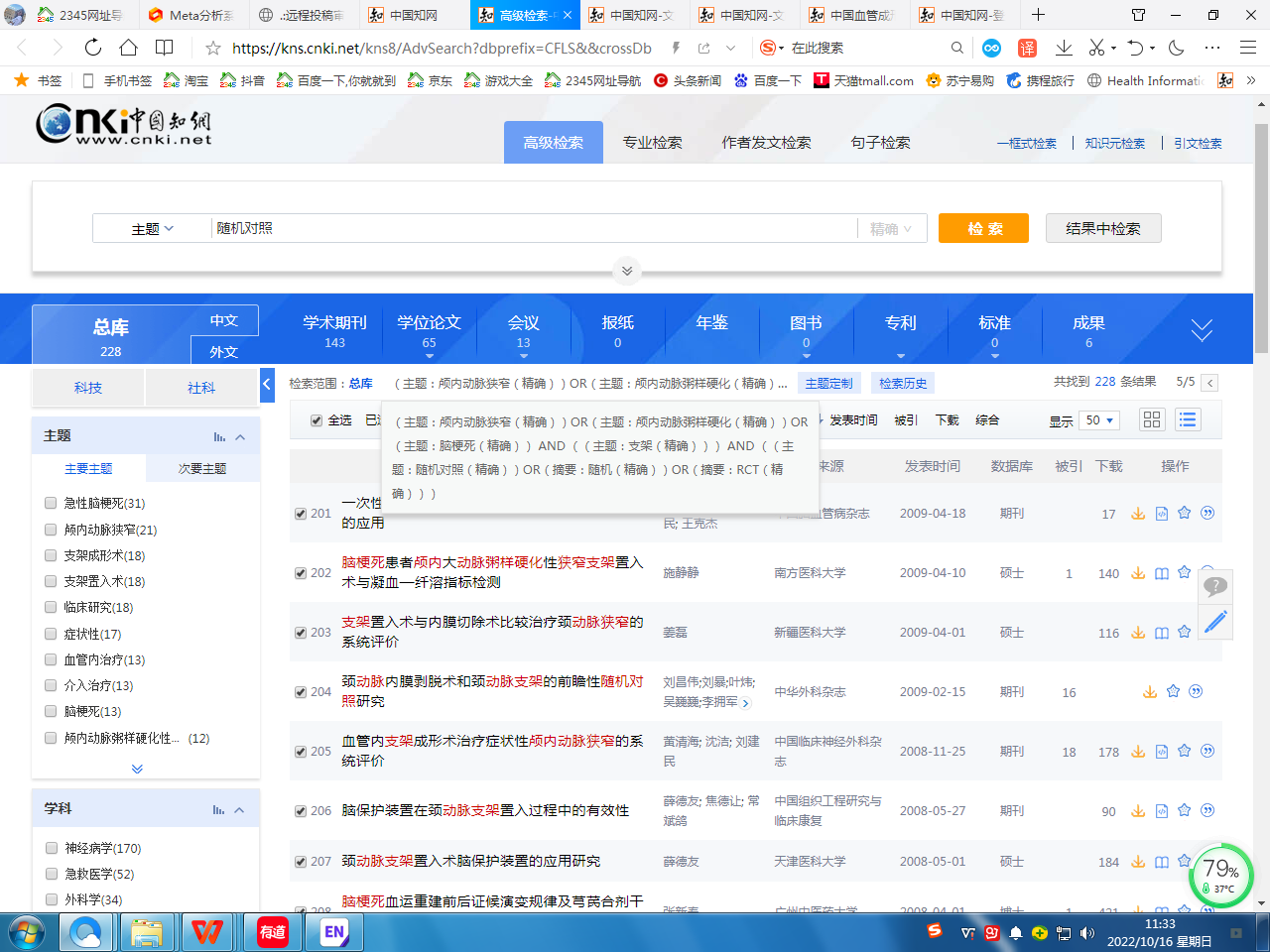

Supplement: Supplementary file 1 [file Table1.DOCX]
